# ERK1/2 signaling induces skeletal muscle slow fiber-type switching and reduces muscular dystrophy disease severity

**DOI:** 10.1172/jci.insight.127356

**Published:** 2019-05-16

**Authors:** Justin G. Boyer, Vikram Prasad, Taejeong Song, Donghoon Lee, Xing Fu, Kelly M. Grimes, Michelle A. Sargent, Sakthivel Sadayappan, Jeffery D. Molkentin

**Affiliations:** 1 Division of Molecular and Cardiovascular Biology, Heart Institute, Cincinnati Children’s Hospital Medical Center, Cincinnati, Ohio, USA.; 2 Heart Lung Vascular Institute, Department of Internal Medicine, University of Cincinnati, Cincinnati, Ohio, USA.; 3 AgCenter, School of Animal Sciences, Louisiana State University, Baton Rouge, Louisiana, USA.; 4 Cincinnati Children’s Hospital Medical Center, Howard Hughes Medical Institute, Cincinnati, Ohio, USA.

**Keywords:** Muscle Biology, Mouse models, Neuromuscular disease, Skeletal muscle

## Abstract

MAPK signaling consists of an array of successively acting kinases. ERK1 and -2 (ERK1/2) are major components of the greater MAPK cascade that transduce growth factor signaling at the cell membrane. Here, we investigated ERK1/2 signaling in skeletal muscle homeostasis and disease. Using mouse genetics, we observed that the muscle-specific expression of a constitutively active MEK1 mutant promotes greater ERK1/2 signaling that mediates fiber-type switching to a slow, oxidative phenotype with type I myosin heavy chain expression. Using a conditional and temporally regulated Cre strategy, as well as *Mapk1* (ERK2) and *Mapk3* (ERK1) genetically targeted mice, MEK1-ERK2 signaling was shown to underlie this fast-to-slow fiber-type switching in adult skeletal muscle as well as during development. Physiologic assessment of these activated MEK1-ERK1/2 mice showed enhanced metabolic activity and oxygen consumption with greater muscle fatigue resistance. In addition, induction of MEK1-ERK1/2 signaling increased dystrophin and utrophin protein expression in a mouse model of limb-girdle muscle dystrophy and protected myofibers from damage. In summary, sustained MEK1-ERK1/2 activity in skeletal muscle produces a fast-to-slow fiber-type switch that protects from muscular dystrophy, suggesting a therapeutic approach to enhance the metabolic effectiveness of muscle and protect from dystrophic disease.

## Abstract 2

ERK1/2 signaling protects muscle from dystrophic disease and induces a slow oxidative fiber-type switch.

## Introduction

Myofibers are individual contractile units that compromise all muscles. They are grossly categorized as type I slow-twitch oxidative myofibers, type IIA fast-twitch oxidative-glycolytic myofibers, or type IIB/IIX fast-twitch glycolytic myofibers (1). These fiber types have distinct molecular and functional properties and can be identified at the histological level by expression of specific myosin heavy chain (MyHC) isoforms and selected metabolic genes. For example, as an adaptation to continual usage, type I fibers express slow-twitch–specific contractile proteins, such as the *Myh7* gene product, and are rich in myoglobin and mitochondria to produce a fatigue resistance profile compared with fast-twitch fibers that are more specialized for bursts of activity (1). The desire to better understand the molecular mechanisms regulating fast-to-slow myofiber switching has been fueled by the potential therapeutic value of a more oxidative metabolic state in chronic diseases such as obesity and type 2 diabetes mellitus (2).

Muscular dystrophies (MDs) consist of a group of inherited disorders characterized by muscle degeneration leading to progressive muscle weakness and ultimately death. A therapeutic strategy currently being studied for MD involves promoting the slow, oxidative phenotype in diseased skeletal muscles because type I fibers appear to protect skeletal muscle from the progression of Duchenne MD (3, 4). Potent regulators of the slow, oxidative program include the calcineurin-signaling pathway (5, 6), the AMPK (7), the PPAR β/δ pathway (8), as well as the transcriptional coactivator peroxisome proliferator-activated receptor γ coactivator 1-α (PGC-1α) pathway (9).

MAPKs are part of a highly conserved network that transduce extracellular signals into an intracellular response involving 3 to 4 tiers of kinases that constitute specific amplifying phosphorylation cascades. The cascade culminates in the phosphorylation and activation of effector kinases, p38, JNK1/2, and ERK1 and -2 (ERK1/2) (10). In ERK1/2 activation, the GTPase Ras at the cell membrane leads to activation of the Raf-1 kinase, which then activates MEK1/2, which are dedicated to ERK1/2 phosphorylation and activation (11, 12). ERK1/2 have conventionally been associated with regulating cell proliferation and cell survival (12); however, in postmitotic differentiated cells the role of ERK1/2 can vary. In cardiomyocytes for example, ERK1/2 signaling regulates eccentric versus concentric cardiac growth (13, 14).

In skeletal muscle, a correlation between exercise and ERK1/2 activation exists. In a study involving human subjects, a one-legged exercise protocol increased ERK1/2 activation relative to the contralateral rested leg (15), whereas marathon runners showed increased ERK1/2 phosphorylation in their muscles (16). With respect to ERK1/2 manipulation in vivo, Shi et al. transfected a plasmid expressing MAPK phosphatase-1 (MKP-1, also known as DUSP1) into the gastrocnemius of mice, which showed an increase in type I fibers, suggesting that inhibition of ERK1/2 causes a fast-to-slow fiber-type conversion (17). However, MKP-1 is not specific to ERK1/2 because it also inhibits JNK1/2 and p38 when overexpressed in vivo (18). Activation of both p38 and JNK MAPKs are also observed following bouts of acute exercise or marathon running, suggesting that these kinases play a role (19). Pogozelski et al. showed that muscle-specific deletion of p38γ in mice attenuated the response to exercise-mediated metabolic adaptations (20). Increasing p38 signaling in skeletal muscle by overexpressing MAPK kinase 6 (MKK6), the upstream kinase responsible for activating p38, resulted in a dystrophic-like phenotype without affecting muscle metabolism (21). Recent data suggest that the increased JNK1/2 expression following exercise is associated with myofiber growth via myostatin/SMAD2 signaling (22). Conversely, the loss of JNK1/2 in myofibers resulted in the presence of smaller muscles with a greater number of oxidative myofibers leading to improved aerobic capacity in mice (22). Here we have taken advantage of mouse genetics to reveal that MEK1, which exclusively activates ERK1/2, leads to induction of the type I, oxidative muscle phenotype in mice. MEK1-ERK1/2 constitutive activation in skeletal muscle also substantially attenuates the severity of MD in a mouse model of this disease.

## Results

### MEK1-ERK1/2 signaling-dependent fast-to-slow fiber-type switch.

**Figure 1:**
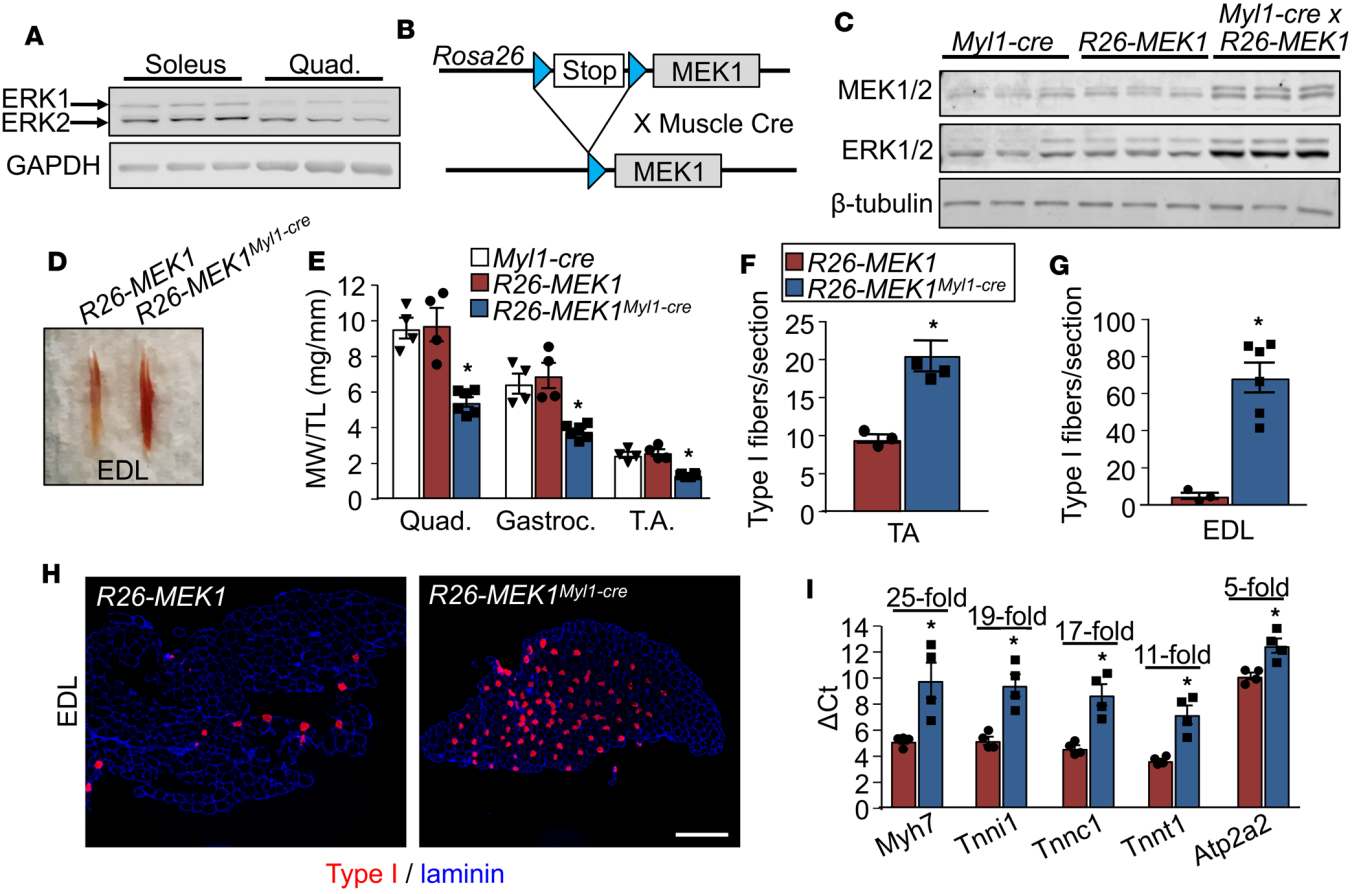
Constitutive active MEK1 expression leads to an increase in type I fibers. (**A**) Western blotting of total ERK1/2 protein in the soleus muscle compared with the quadriceps muscle from 3 independent wild-type animals at 3 months of age. GAPDH is shown as loading control. (**B**) Schematic representation of the constitutively active MEK1 cDNA targeted to the *Rosa26* locus with a stop cassette and flanking loxP sites (blue), whereby Cre recombination gives expression. (**C**) Western blot analysis for the indicated proteins using gastrocnemius (gastroc) lysate from 6-month-old mice of the indicated genotypes. Results from 3 different mice are shown. (**D**) Representative image of the extensor digitorum longus (EDL) muscles from 6-month-old *Rosa26*-*MEK1* and *Rosa26*-*MEK1^Myl1–cre^* mice. (**E**) Relative muscle weights (MW) of quadriceps (quad), gastroc, and tibialis anterior (TA) muscles normalized to tibia length (TL) from 6-month-old *Rosa26-MEK1^Myl1–cre^* mice, *n* = 6; *Myl1-cre*, *n* = 4; and *Rosa26*-*MEK1*, *n* = 4. One-way ANOVA with Tukey’s multiple comparisons test was used to determine significance, *P < 0.05 versus controls. Data are plotted as the mean and the error bars represent SEM. (**F** and **G**) Quantification of slow, oxidative type I fibers across an entire muscle histological section at the mid-belly from the TA and EDL taken from 6-month-old mice of the indicated genotypes. EDL and TA for *Rosa26*-*MEK1*, *n* = 3; EDL for *Rosa26*-*MEK1^Myl1–cre^*, *n* = 6; and TA for *Rosa26*-*MEK1^Myl1–cre^*, *n* = 4. A 2-tailed *t* test was used to analyze groups for statistical significance. *P < 0.05 versus *Rosa26*-*MEK1*. Data represent mean ± SEM. (**H**) Representative histological sections from the EDL muscle immunostained for Myh7 (type I myosin) protein (red) and laminin (blue) from mice of the indicated genotypes. Scale bars: 500 μm. (**I**) mRNA levels for Myh7, troponin I1 (Tnni1), troponin C1 (Tnnc1), troponin T1 (Tnnt1), and sarcoplasmic/endoplasmic reticulum calcium ATPase 2 (Atp2a2) from the EDL muscle from 6-month-old mice of the indicated genotypes. Data are plotted as the mean ΔCt for each genotype and fold changes calculated using the ΔΔCt method are indicated above the results for each transcript tested. Error bars represent SEM. Fold-change ranges are provided in Supplemental Table 1. *n* = 4 for both groups. Significance was determined using a 2-tailed *t* test, *P < 0.05.

Given that ERK1/2 levels are increased in the muscles of marathon runners (16), we hypothesized that this signaling pathway might play a role in promoting a slow, oxidative phenotype. We first observed that total ERK1/2 protein expression is increased in the slow-twitch soleus muscle of the mouse compared with the primarily fast-twitch quadriceps muscle (Figure 1A). To mechanistically evaluate causation, we used a genetic approach in the mouse in which a line containing a constitutively active MEK1 cDNA (*Map2k1* gene product*)* inserted into the *Rosa26* locus containing a Cre-dependent “Stop”-cassette was crossed with various mouse models expressing Cre in muscle (Figure 1B). More specifically, *Rosa26*-*MEK1* mice were crossed with *Myl1*-*cre* gene-targeted mice (Cre inserted into the myosin light chain 1/3 locus) which express Cre-recombinase in differentiating myofibers (23). Doing so resulted in skeletal muscles from *Rosa26*-*MEK1^Myl1–cre^* mice having increased total MEK1 and ERK1/2 expression levels (Figure 1C and Supplemental Figure 1A; supplemental material available online with this article; https://doi.org/10.1172/jci.insight.127356DS1). The increase in total ERK1/2 protein observed in the *Rosa26*-*MEK1^Myl1–cre^* skeletal muscle-specific mice was previously observed in cardiac-specific MEK1-transgenic mice (24). To demonstrate that the constitutively active MEK1 allele led to an increase in ERK1/2 signaling, we interrogated the expression level of known nuclear and cytoplasmic ERK1/2 substrates. Increased expression levels of phosphorylated (active) ETS domain-containing protein (ELK-1) were observed in samples from *Rosa26*-*MEK1^Myl1–cre^* mice compared with controls (Supplemental Figure 1B). Early growth response protein 1 (EGR1) is a transcription factor and a known downstream target of ELK-1 (25, 26). EGR1 levels were also increased in skeletal muscle with greater active MEK1 expression relative to controls suggesting that this branch of the ERK1/2 pathway was active. The expression levels of ribosomal s6 kinase (RSK1) were more abundant in muscle lysate samples from *Rosa26*-*MEK1^Myl1–cre^* mice compared with *Rosa26*-*MEK1* controls. Importantly, in *Rosa26*-*MEK1^Myl1–cre^* muscles, we observed an increase in site-specific phosphorylation in the ERK1/2 substrate RSK1 (residues Th359 and Ser363) (ref. 27 and Supplemental Figure 1B). Once activated by ERK1/2, RSK1 is autophosphorylated at serine 380, which was also increased in the *Rosa26*-*MEK1^Myl1–cre^* muscle samples compared with controls (27). Similarly, for mitogen- and stress-activated kinase 1 (MSK1), another ERK1/2 substrate, we observed an increase in total MSK1 levels as well as the phosphorylated (Th581) form of the protein in *Rosa26*-*MEK1^Myl1–cre^* lysates samples relative to controls (Supplemental Figure 1B). ERK1/2 phosphorylation of MSK1 precedes the autophosphorylation of the linker region at multiple sites, including serine 376, which was also increased in the *Rosa26*-*MEK1^Myl1–cre^* samples (27). Collectively, the increase in phosphorylated status of previously identified ERK1/2 substrates demonstrated activation of the ERK1/2 MAPK pathway in skeletal muscle from *Rosa26*-*MEK1^Myl1–cre^* mice.

Skeletal muscle from *Rosa26-MEK1^Myl1–cre^* mice, such as the extensor digitorum longus (EDL), showed a remarkably prominent increase in red coloration compared with the *Rosa26-MEK1* only controls, suggesting a slow, oxidative program switch (Figure 1D). This difference was evident both at age 2 months and 6 months (Figure 1D and Supplemental Figure 1C). Relative muscle weights from the quadriceps, gastrocnemius, and tibialis anterior (TA) muscles were also substantially reduced in *Rosa26-MEK1^Myl1–cre^* mice compared with controls at age 2 months (Supplemental Figure 1D) and these were even more pronounced at age 6 months (Figure 1E).

Muscle histological sections were collected and stained for type I myosin heavy chain (*Myh7* gene product) to identify fibers that were associated with the slow-twitch program (the number of type I positive fibers was counted per muscle section). Compared with *Rosa26*-*MEK1* controls, we observed a substantial increase in type I fibers in the TA, the soleus, and the EDL (Figure 1, F–H, and Supplemental Figure 1, E–H). To support these histological results, quantitative real-time PCR (qPCR) was used to assess mRNA expression of genes preferentially expressed in slow-twitch myofibers, which showed a dramatic shift in gene expression toward the slow, oxidative fiber type in EDL muscle from *Rosa26-MEK1^Myl1–cre^* mice versus controls (Figure 1I). Despite these robust expression changes of slow muscle genes, the mRNA and protein expression of PGC1α and of PPARγ remained unchanged between groups (data not shown).

### ERK2 underlies the slow-twitch phenotype observed downstream of MEK1.

**Figure 2:**
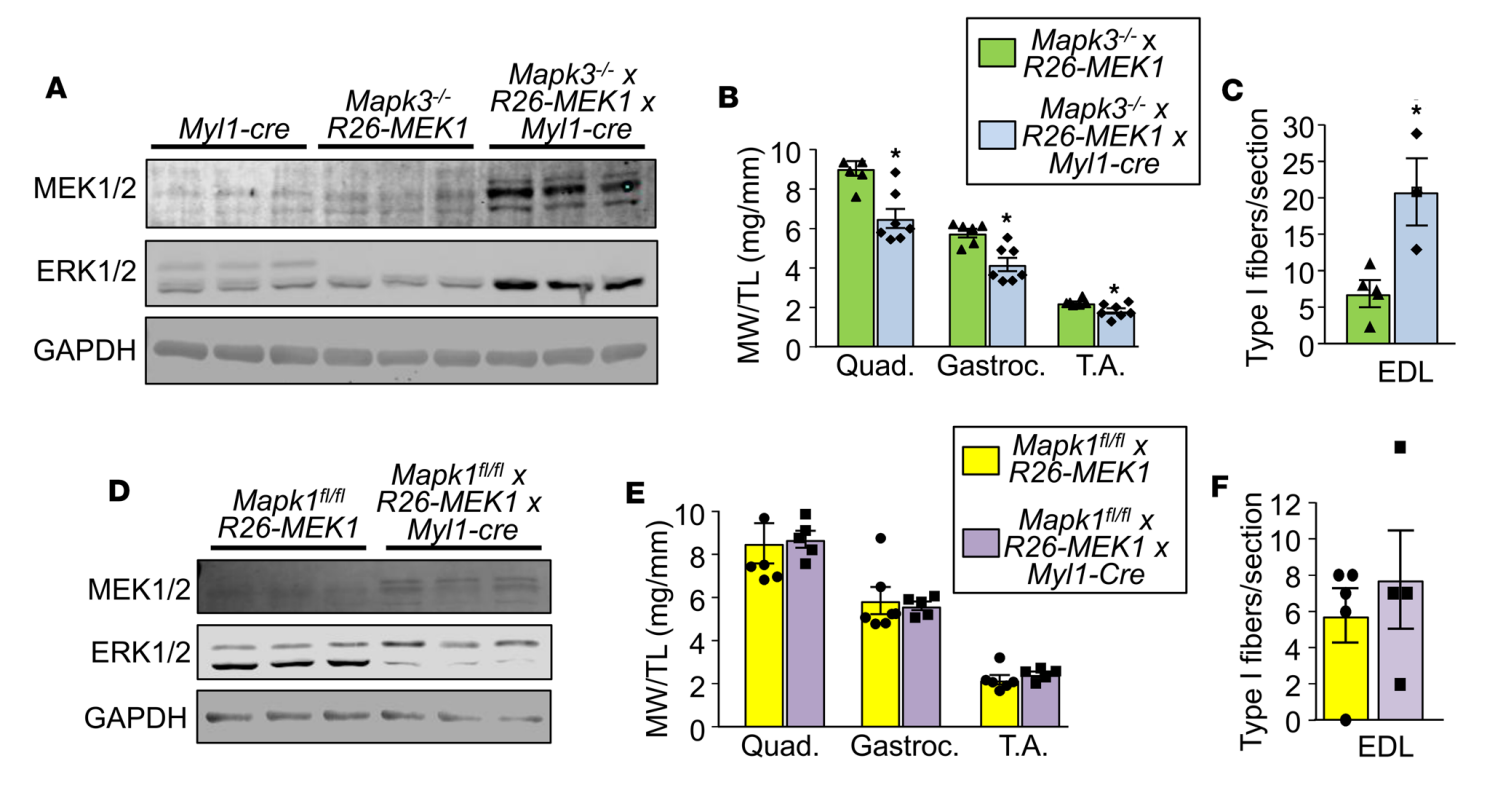
ERK2 drives the MEK1-mediated fast-to-slow fiber-type switch. (**A**) Western blot analysis for total MEK1/2 and ERK1/2 protein using lysate from the gastroc muscle of 5-month-old mice of the indicated genotypes. *n* = 3 for both groups. GAPDH is shown as a loading control. (**B**) Relative muscle weight/tibia length (MW/TL) at 5 months of age for the quad, gastroc, and TA muscle from mice of the indicated genotypes. *n* = 6, *Mapk3^–/–^* × *Rosa26*-*MEK1*; *n* = 7, *Mapk3^–/–^* × *Rosa26*-*MEK1^Myl1–cre^*. A 2-tailed *t* test was used to analyze groups for statistical significance, *P < 0.05 versus controls. Data represent mean ± SEM. (**C**) Quantification of total type I fibers in a histological section at the mid-belly of the EDL muscle from mice of the indicated genotypes. *n* = 4, *Mapk3^–/–^* × *Rosa26*-*MEK1*; *n* = 3, *Mapk3^–/–^* × *Rosa26*-*MEK1^Myl1–cre^*. Statistical significance was determined using a 2-tailed Student’s *t* test. *P < 0.05 versus control. Data represent mean ± SEM. (**D**) Western blot analysis for total MEK1/2 and ERK1/2 protein using lysates from the gastroc muscle from 5-month-old mice of the indicated genotypes. GAPDH was used a loading control. *n* = 3 for both groups. (**E**) MW/TL at 5 months of age for the quad, gastroc, and TA muscle from mice of the indicated genotypes. *n* = 6, *Mapk1^fl/fl^* × *Rosa26*-*MEK1; n* = 5, *Mapk1^fl/fl^* × *Rosa26*-*MEK1^Myl1–cre^*. Data represent mean ± SEM. (**F**) Quantification of total type I fibers in a muscle histological section taken at the mid-belly of the EDL muscle from mice of the indicated genotypes. *n* = 5, *Mapk1^fl/fl^* × *Rosa26*-*MEK1*; *n* = 4, *Mapk1^fl/fl^* x *Rosa26*-*MEK1^Myl1–cre^*. Data represent mean ± SEM.

We have previously observed a greater need for ERK2 protein (*Mapk1* gene) relative to ERK1 protein (*Mapk3* gene) in the heart in programming adaptive responsiveness (28). We evaluated whether ERK1 or ERK2 preferentially contributed to the fast-to-slow fiber-type switch observed in the activated MEK1-expressing mice. We crossed *Rosa26*-*MEK1* and *Myl1*-cre alleles into the *Mapk1* loxP (fl) site-targeted background (29) or separately into the germline *Mapk3^–/–^* background (30). Loss of *Mapk3* gave a compensatory increase in ERK2 protein expression in the presence of the activated *Rosa26*-MEK1–expressing allele (Figure 2A), although it still caused a decrease in skeletal muscle weights of the quadriceps, gastrocnemius, and TA (Figure 2B), and a large increase in type I fibers (Figure 2C). For the second cross, the *Myl1-cre* produces recombination of both the *Rosa26*-*MEK1* and *Mapk1^f/f^* alleles in myofibers. These mice showed increased MEK1 expression and reduced ERK2 protein (bottom band) with a mild increase in ERK1 protein (top band, Figure 2D). Skeletal muscle weights in the quadriceps, gastrocnemius, and TA were unchanged between normal controls and MEK1-expressing mice lacking *Mapk1* in muscle (Figure 2E). More importantly, loss of *Mapk1* from muscle now eliminated the ability of MEK1 to augment type I positive myofiber number compared with controls (Figure 2F). These results demonstrate that ERK2 is the primary downstream MEK1 effector responsible for skeletal muscle fiber-type switching in mice. As an aside, ERK1/2 double-deleted mice consisting of *Mapk3^–/–^* *Mapk1^fl/fl–Myl1–cre^* alleles were extremely runted and none lived past 30 days so they could not be reliably analyzed (data not shown).

### MEK1 drives the slow, oxidative program in myoblast progenitors and adult muscle.

We crossed the *Rosa26*-*MEK1* allele-containing mice with mice containing the *MyoD-icre* targeted allele (31). This strategy allowed us to induce MEK1-ERK1/2 signaling at an early stage in muscle progenitors of the developing embryo, irrespective of initial fiber type (31). Skeletal muscle from these mice showed a marked increase in total MEK1 and ERK1/2 protein (Supplemental Figure 2A), similar to the increases observed in skeletal muscles from *Rosa26-MEK1^Myl1–cre^* mice. At age 6 months, relative muscle weights were also substantially decreased in *Rosa26*-*MEK1^MyoD–icre^* mice compared with controls (Supplemental Figure 2B), and at the histological level, the number of type I myofibers was also notably increased in the soleus and EDL muscles compared with *Rosa26*-*MEK1* littermate controls (Supplemental Figure 2, C–E).

**Figure 3:**
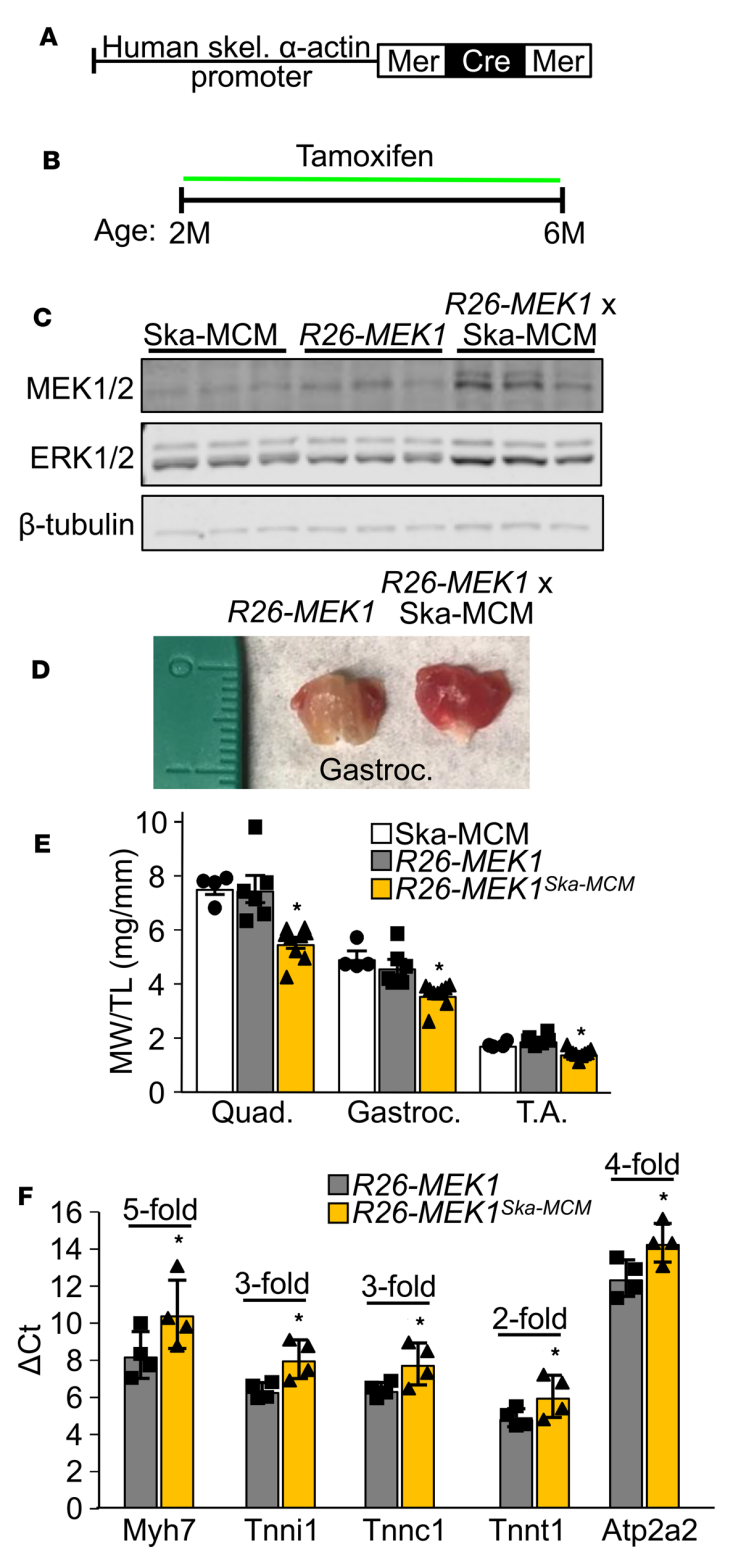
Induction of activated MEK1 expression in mature myofibers induces a slow, oxidative phenotype. (**A**) Schematic of the MerCreMer cDNA driven by the human skeletal α-actin promoter in generating tamoxifen inducible and muscle-specific transgenic mice. (**B**) Tamoxifen dosing regimen to induce constitutive active MEK1 expression for the experiments shown in this figure. (**C**) Western blot analysis for total MEK1/2 and ERK1/2 protein using lysate from the gastroc muscle of mice of the indicated genotypes. *n* = 3 per group. β-Tubulin is shown as a loading control. (**D**) Representative image of the gastroc muscle from 6-month-old mice of the indicated genotypes. (**E**) Relative muscle weight/tibia length (MW/TL) at 6 months of age for the quad, gastroc, and TA from Ska-MCM (*n* = 4), *Rosa26*-*MEK1* (*n* = 6), and *Rosa26*-*MEK1^Ska–MCM^* (*n* = 9) mice. One-way ANOVA with Tukey’s multiple comparisons test was used for statistical analysis. *P < 0.05 versus controls. Data represent mean ± SEM. (**F**) mRNA levels determined by qPCR for the indicated genes from the gastroc muscle of 6-month-old mice of the indicated genotypes. Data are presented as the mean ΔCt for each group, error bars represent SEM. Fold differences calculated using the ΔΔCt method are indicated for each transcript tested. *n* = 4 per group. Significance was determined using a 1-tailed Student’s *t* test, *P < 0.05. Fold-change ranges are provided in Supplemental Table 2.

To determine whether a greater slow fiber phenotype could be induced in adult mice, we used a transgene in which the human skeletal α-actin promoter drives the tamoxifen-regulated MerCreMer cDNA (Ska-MCM, Figure 3A and ref. 32). We began administering tamoxifen to *Rosa26*-*MEK1^Ska–MCM^* mice as well as to control *Rosa26*-*MEK1* littermates and Ska-MCM control mice at age 2 months (Figure 3B). By 6 months, Western blot analysis again revealed an increase in total MEK1 and ERK1/2 proteins in skeletal muscle from *Rosa26*-*MEK1^Ska–MCM^* mice compared with controls (Figure 3C), and more importantly, a dramatic increase in the slow, oxidative program and a concomitant decrease in muscle weights (Figure 3, D and E). qPCR analysis revealed an increase in mRNA expression of cytoskeletal markers of slow twitch muscle, namely, *Myh7*, the slow muscle troponins, as well as the *Atp2a2* gene (SERCA2a) in mice after MEK1 induction (Figure 3F). These results indicate that adult myofibers retain the capacity to respond to MEK1-ERK1/2 signaling in switching to a slow, oxidative myofiber program.

**Figure 4:**
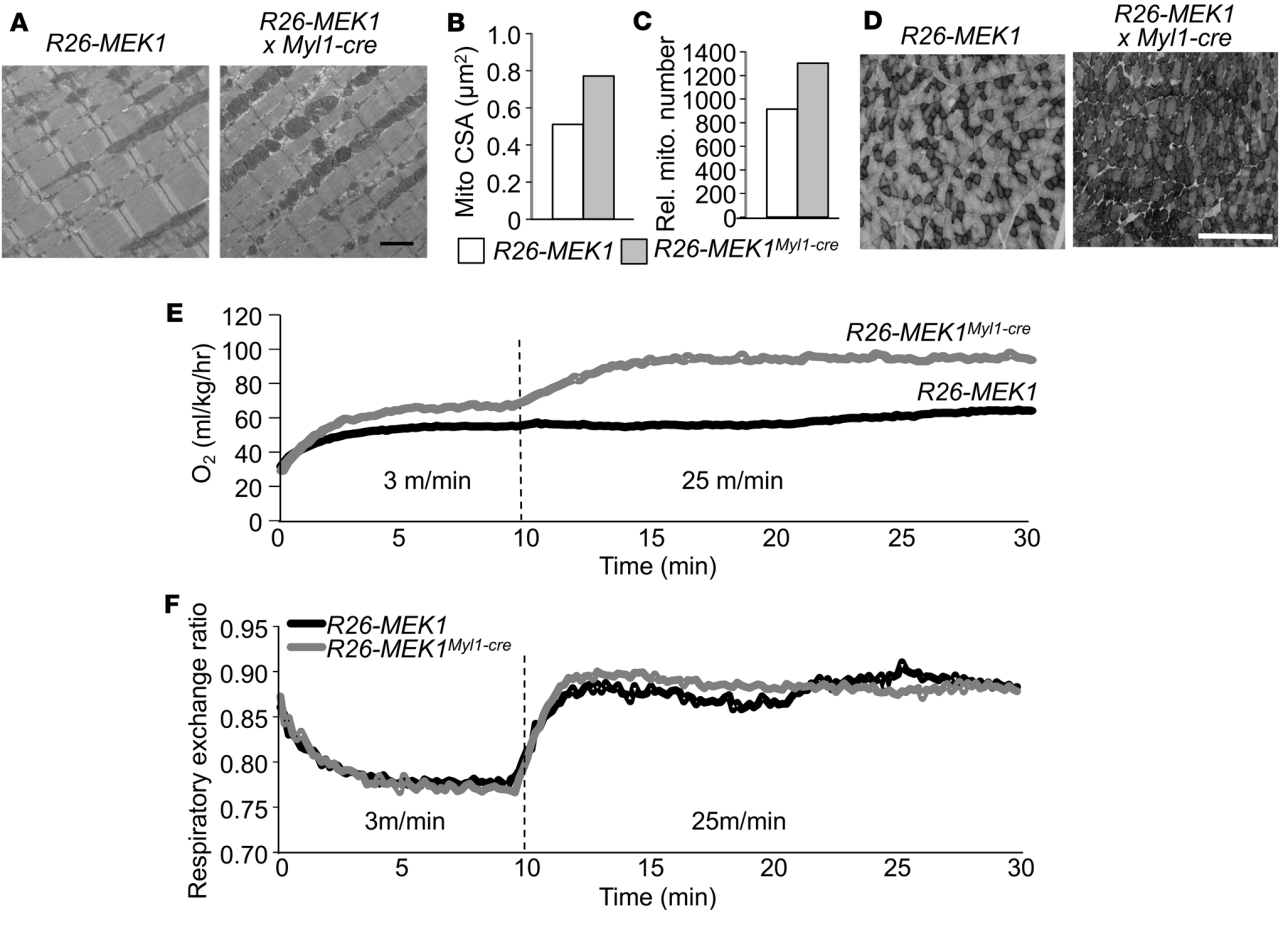
Increased oxygen consumption in *Rosa26*-*MEK1^Myl1–cre^* mice. (**A**) Representative electron micrographs of longitudinal histological sections from the TA of 2-month-old *Rosa26*-*MEK1* and *Rosa26*-*MEK1^Myl1–cre^* mice. Scale bar: 2 μm. (**B**) Quantification of mitochondria cross-sectional area (CSA) from 2-month-old mice of the indicated genotypes; *n* = 2 per group. (**C**) Total mitochondria number quantified from images as shown in **A**, from *Rosa26*-*MEK1* and *Rosa26*-*MEK1^Myl1–cre^* mice at 2 months of age; *n* = 2 per group. (**D**) Representative images of SDH-stained histological sections taken at the mid-belly of the gastroc from 6-month-old mice of the indicated genotypes. The darker stained fibers show SDH reactivity. Scale bar: 500 μm. (**E**) Oxygen consumption and (**F**) respiratory exchange ratio from 2-month-old *Rosa26*-*MEK1* and *Rosa26*-*MEK1^Myl1–cre^* mice during treadmill acclimatization (3 m/min) and exercise (25 m/min). *n* = 5 for both groups. Data are plotted as the mean for **B**–**F**.

*Increased mitochondria and oxygen consumption in Rosa26-MEK1^Myl1–cre^animals*. To further define the presumably enhanced oxidative muscle phenotype in *Rosa26*-*MEK1^Myl1–cre^* mice, we quantified mitochondrial content and architecture by electron microscopy of the TA muscle at age 2 months. Mitochondria from *Rosa26-MEK1^Myl1–cre^* mice were larger than those from *Rosa26*-*MEK1* control mice (Figure 4, A and B), and there were higher numbers of them (Figure 4C). Muscle sections from the gastrocnemius muscle of *Rosa26-MEK1^Myl1–cre^* mice showed increased staining for the mitochondrial enzyme succinate dehydrogenase, a result indicative of greater oxidative respiration capacity (Figure 4D).

Type I fibers are known to consume more oxygen; therefore, we performed indirect calorimetry in 2-month-old *Rosa26-MEK1^Myl1–cre^* mice. Mice were housed in a sealed container with a motorized treadmill apparatus with an Oxymax system for gas analysis. Animals were acclimatized to the treadmill at the lowest speed setting (3 m/min) for 10 minutes. During this initial period, we observed an increase in oxygen consumption in *Rosa26-MEK1^Myl1–cre^* mice compared with controls (Figure 4E). Mice were then sprinted, and we observed an even greater oxygen consumption differential between *Rosa26-MEK1^Myl1–cre^* mice and controls (Figure 4E). We also measured the respiratory exchange ratio (RER) to infer metabolic substrate utilization. RER values of 1 indicate preferential glucose oxidation, whereas RER values of 0.7 indicate preferential fatty acid oxidation. No differences in substrate utilization between *Rosa26-MEK1^Myl1–cre^* and control mice were observed during the exercise protocol (Figure 4F). Taken together, these results are consistent with a shift in all musculature toward a slow, oxidative fiber phenotype that is the result of augmented MEK1-ERK1/2 signaling.

### TA muscles from Rosa26-MEK1^Myl1–cre^ mice are fatigue resistant.

**Figure 5:**
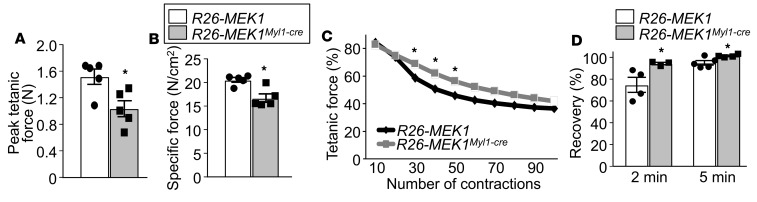
Skeletal muscles from *Rosa26*-*MEK1^Myl1–cre^* mice are fatigue resistant. (**A**) Peak tetanic force and (**B**) normalized specific force were measured from the TA muscle of 4-month-old *Rosa26*-*MEK1* and *Rosa26*-*MEK1^Myl1–cre^* mice; *n* = 5 per group. Data represent mean ± SEM. Significance was determined using a 2-tailed Student’s *t* test, *P < 0.05. (**C**) TA muscles from *Rosa26*-*MEK1* and *Rosa26*-*MEK1^Myl1–cre^* mice were contracted 100 times to elicit fatigue. Peak tetanic forces are expressed as percentage of the prefatigue peak tetanic force. *n* = 4 (*Rosa26*-*MEK1*) and *n* = 5 (*Rosa26*-*MEK1^Myl1–cre^*). Significance was determined using a 1-tailed *t* test, *P < 0.05. (**D**) Peak tetanic force was recorded 2 minutes and 5 minutes after fatigue to assess recovery in the TA muscles from the indicated groups of mice. *n* = 4 (*Rosa26*-*MEK1*) and *n* = 5 (*Rosa26*-*MEK1^Myl1–cre^*). Data are presented as percentage of the prefatigue peak tetanic force and the error bars represent SEM. Significance was determined using a 2-tailed *t* test, *P < 0.05.

To evaluate how increased MEK1-ERK1/2 signaling impacted physiologic performance, we evaluated in situ contractile function of the TA muscle in 4-month-old *Rosa26-MEK1^Myl1–cre^* and *Rosa26*-*MEK1* control mice. We observed a substantial decrease in absolute maximal peak isometric tetanic force and the size-normalized specific force produced by the TA muscle from *Rosa26-MEK1^Myl1–cre^* mice compared with controls (Figure 5, A and B). These results agree with those previously reported in muscles from other mouse models rich in type I fibers, which show less maximal force generation capacity (33). However, when subjected to a fatigue protocol in which we elicited 100 muscle contractions with only 3 seconds between each contraction, muscle from *Rosa26-MEK1^Myl1–cre^* mice showed greater maintained force compared with *Rosa26-MEK1* control mice (Figure 5C). Following the fatigue protocol, we again measured the maximal force produced by both groups at two different time points to assess force recovery. At both 2 and 5 minutes following the fatigue protocol, the TA muscle from *Rosa26-MEK1^Myl1–cre^* mice still showed substantially greater recovery of maximal force generation compared with *Rosa26-MEK1* controls (Figure 5D). These data demonstrate that increased MEK1-ERK1/2 signaling produces muscle that is more resistant to fatigue.

### Additional genetic approaches to alter ERK1/2 signaling in skeletal muscle.

The *Rosa26-MEK1^Myl1–cre^* model results in constitutive expression of an active MEK1 mutant in skeletal muscle, which is not entirely physiologic. Therefore, we also analyzed *Dusp6/8* double-null mice, which we previously showed that each single null had increased ERK1/2 activity in the heart, but within more physiologic parameters (34, 35). Dual-specificity protein phosphatases (DUSP) are capable of directly binding to the activation loop of MAPK effectors, including ERK1/2 leading to their dephosphorylation and inactivation (36). Thus, loss of select DUSP proteins leads to augmented MAPK activity. In addition, DUSP6 was shown to be a key modulator of ERK1/2 signaling in skeletal muscle (37).

Comparable to what we observed with expression of activated MEK1 in myofibers (Figure 1D), skeletal muscles from *Dusp6/8^–/–^* mice also appeared redder in color relative to wild-type age-matched controls, suggesting a shift toward the slow, oxidative phenotype (Supplemental Figure 3A). At age 4 months, histological sections from the TA muscles of *Dusp6/8^–/–^* mice showed substantially more type I myofibers compared with control TA muscle (Supplemental Figure 3, B and C). We also observed a decrease in Myh4-positive myofibers, a marker of type IIB fibers, in *Dusp6/8^–/–^* histological samples relative to controls (Supplemental Figure 3, B and D). These results suggest that enhanced ERK1/2 signaling caused by the absence of critical DUSPs leads to an oxidative phenotype reminiscent to the *Rosa26*-*MEK1^Myl1–cre^* model. This finding also highlights DUSP6 and DUSP8 as relevant therapeutic targets to promote an oxidative phenotype in muscle.

Finally, we also used a Myh7 (type I myosin) promoter-driven Cre mouse line (6) to delete *Mapk1* and *Mapk3* in existing slow muscle fibers, of which the soleus muscle has the largest density. We observed the complete absence of ERK1 and a reduction in ERK2 protein levels in soleus muscle samples from *Mapk3^–/–^ Mapk1*^fl/fl–Myh7–cre^ compared with the Myh7-cre transgene-only control mice (Supplemental Figure 4A). The reduction in ERK2 protein levels coincided with the proportion of type I fibers present in the soleus muscle (~45%) (Supplemental Figure 4A). Immunohistochemistry from soleus muscle in 4-month-old *Mapk3*^–/–^; *Mapk1*^fl/fl–Myh7–cre^ mice showed ectopic expression of Myh2 in type I slow fibers, therefore creating type I-IIA hybrid fibers that were extremely rare in controls (Supplemental Figure 4, B and C). These data suggest that not only can ERK1/2 signaling induce a slow, oxidative phenotype in skeletal muscles, its activity is required for aspects of slow fiber type specification.

### MEK1-ERK1/2 signaling reduces muscle damage in a mouse model of muscular dystrophy.

**Figure 6:**
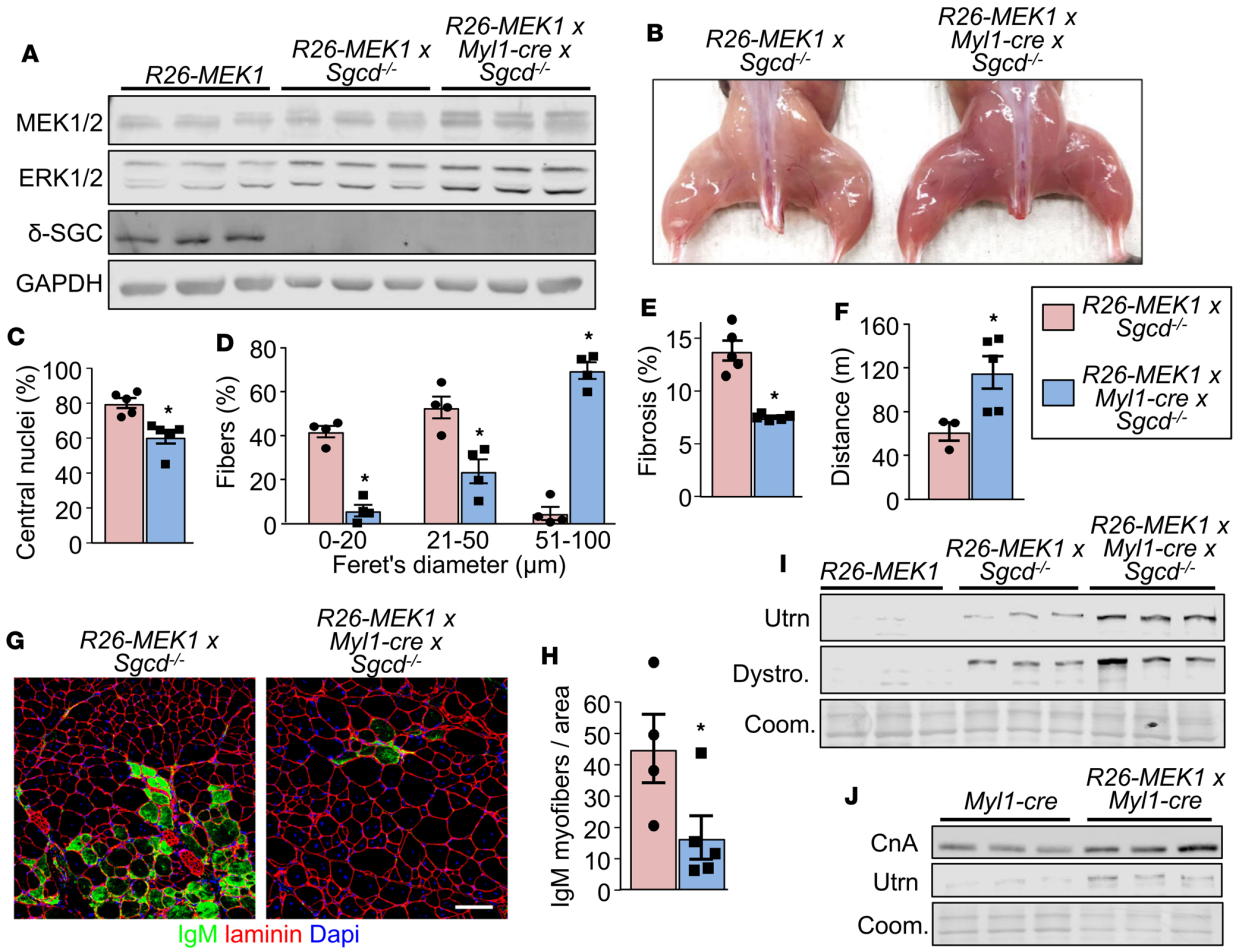
MEK1-ERK1/2 signaling protects myofibers from muscular dystrophy. (**A**) Western blot for total MEK1/2, ERK1/2 and δ-sarcoglycan (δ-SGC) protein levels from muscle protein lysates of mice from the indicated genotypes at 3 months of age. *n* = 3 for all groups. GAPDH is shown as loading control. (**B**) Representative image of the hindlimb muscles from 3-month-old mice of the indicated genotypes. (**C**) Quantification of myofibers with centrally located nuclei in histological sections of the quad from 3-month-old mice of indicated genotypes; *n* = 5 per group. Significance was determined using a 2-tailed Student’s *t* test. *P < 0.05. Average values are presented as a percentage from all fibers analyzed, error bars represent SEM. (**D**) Fiber size distribution quantified from the quad of mice of the indicated genotypes at 3 months of age. The mean (±SEM) percentage value relative to all fibers analyzed was graphed, *n* = 4 per group. Significance was determined using a 2-tailed Student’s *t* test. *P < 0.05. (**E**) Quantification of interstitial fibrosis assessed by picrosirius red staining of quad muscle histological sections from 3-month-old mice of the indicated genotypes. *n* = 5 per group. Significance was determined using a 2-tailed Student’s *t* test. *P < 0.05. (**F**) Average time spent running on a treadmill with 3-month-old mice of the indicated genotypes. *n* = 3 (*Rosa26*-*MEK1 Sgcd^–/–^)* and *n* = 5 (*Rosa26*-*MEK1^Myl1–cre^* *Sgcd^–/–^*). Significance was determined using a 2-tailed Student’s *t* test. *P < 0.05. (**G**) Representative immunohistochemical images showing myofibers stained with immunoglobulin M (IgM) antibody (green) and with laminin antibody (red) to delineate the myofibers. Images from the quad are shown from the indicated genotypes of mice at 3 months of age. Scale bars: 100 μm. (**H**) IgM positive myofibers quantification from histological sections as shown in **G**. Data are presented as the mean number of IgM positive fibers for a given area. Error bars represent SEM; *n* = 5 per group. Significance was determined using a 2-tailed Student’s *t* test. *P < 0.05. (**I**) Western blot for utrophin A (Utrn) and dystrophin (Dystro) using gastroc protein lysate from 3-month-old mice of the indicated genotypes. Results from 3 different mice are shown. Coomassie (Coom) staining was used to show equal loading. (**J**) Western blot analysis for calcineurin A (CnA) and Utrn using gastroc muscle of mice of the indicated genotypes at 6 months of age. Equal loading was assessed using Coom stain. Results from 3 separate mice are shown.

Slow myofibers appear to be more resistant to MD compared with fast-glycolytic fibers (38), suggesting a therapeutic vantage point (39). Here we crossbred the *Rosa26*-*MEK1^Myl1–cre^* mice with δ-sarcoglycan-null (*Sgcd^–/–^*) mice, which models limb-girdle muscular dystrophy type 2F (40). Western blot analysis confirmed an increase in total MEK1 and ERK1/2 protein levels and δ-sarcoglycan deletion in gastrocnemius muscle samples from 3-month-old *Rosa26*-*MEK1^Myl1–cre^* *Sgcd^–/–^* animals compared with controls (Figure 6A), along with an increase in slow, oxidative muscle (Figure 6B). We next assessed several pathological indices in the quadriceps, which was selected because it is more affected than other hindlimb muscles in *Sgcd*^–/–^ animals. Fewer myofibers with centrally located nuclei were observed in *Sgcd^–/–^* animals with increased ERK1/2 signaling (Figure 6C). A dramatic increase in the size of the myofibers that were spared from degeneration was also observed in histological sections taken from *Rosa26*-*MEK1^Myl1–cre^* *Sgcd^–/–^* muscle compared with *Rosa26*-*MEK1* *Sgcd*^–/–^ controls (Figure 6D). Interstitial fibrosis in the quadriceps was also reduced in *Rosa26*-*MEK1^Myl1–cre^* *Sgcd^–/–^* mice compared with *Rosa26*-*MEK1* *Sgcd*^–/–^ control mice at age 3 months (Figure 6E). From a functional standpoint, *Rosa26*-*MEK1^Myl1–cre^* *Sgcd^–/–^* mice ran further in a forced treadmill protocol compared with *Rosa26*-*MEK1* *Sgcd*^–/–^ control mice (Figure 6F). We also examined the stability of the myofiber sarcolemma in the quadriceps by immunostaining for the presence of immunoglobulin M (IgM) inside myofibers. Fewer IgM positive myofibers were observed in histological sections from mice with increased ERK1/2 signaling compared with controls, suggesting that the underlying membrane defect from δ-sarcoglycan deficiency is partially compensated (Figure 6, G and H).

To possibly explain why increased ERK1/2 activity was protective to MD in *Sgcd^–/–^* mice, we observed that both utrophin A (39) and dystrophin were upregulated in expression beyond the levels observed in *Rosa26-MEK1* control mice and even above the level of existing compensation because of loss of δ-sarcoglycan (Figure 6I). Utrophin mRNA expression has previously been reported to correlate with oxidative capacity of skeletal muscle in a calcineurin signaling–dependent manner (41, 42). Thus, we examined whether the reduced muscle damage caused by increased ERK1/2 activity and increased utrophin A/dystrophin was also associated with increased calcineurin signaling. The data show that even in nondystrophic *Rosa26*-*MEK1^Myl1–cre^* mice, there is induction of utrophin A, as well as calcineurin A levels in the gastrocnemius muscle (Figure 6J). These results suggest that the calcineurin/NFAT signaling axis, which is known to also program slow, oxidative skeletal muscle (43, 44), is likely contributing to the utrophin A upregulation in protecting *Sgcd*^–/–^ mice from MD.

## Discussion

A proteomic screen revealed that some 562 proteins become phosphorylated in human skeletal muscle following exercise (45), suggesting complex adaptive kinase-dependent signaling responsiveness. In the present study, we identify a role for MAPK signaling in promoting a fast-to-slow fiber-type transition under basal conditions in vivo. The MEK1-ERK1/2 signaling pathway produced a greater number of slow, oxidative fibers in skeletal muscle, which now consumes more oxygen and is more fatigue resistant compared with fast-glycolytic fibers.

ERK1 and ERK2 share 85% amino acid sequence identity, and each appears to phosphorylate the same substrates in vitro (46). Despite their sequence similarity, the requirement for ERK1 versus ERK2 during development differs substantially. Whereas MEK2 and ERK1 are dispensable for embryonic development, studies from gene-deleted mice revealed that MEK1 and ERK2 are essential for development because loss of either gene product results in embryonic lethality (47). We demonstrate here that MEK1-mediated activation of ERK2 but not ERK1 was required for this slow, oxidative phenotypic switch in muscle. However, we believe this simply reflects a greater abundance of ERK2 in skeletal muscle relative to ERK1, especially given the very high levels of ERK2 upregulation observed in *Mapk3*-null muscle, whereas ERK1 protein levels were only mildly upregulated in *Mapk1*-deleted muscle (Figure 2, A and D). This same observation was made in the heart, in which ERK2 levels predominated and resulted in a greater functional effect over ERK1 (13).

Our observations that MEK1-ERK1/2 signaling augments the slow, oxidative fiber-type program are actually opposite of the interpreted results of a previous report (17). Shi et al. transfected adult skeletal muscle with an MKP-1–expressing plasmid, which showed increased type IIA and type I fibers, indicating a more slow, oxidative program with ERK1/2 dephosphorylation (17). However, MKP-1 is actually more specific for p38 MAPK and it can dephosphorylate all 3 major terminal MAPKs when overexpressed (18). In our study, we used multiple muscle-specific Cre drivers to induce MEK1 activation during myogenesis and in adulthood, as well as *Dusp6/8* double-null mice as another independent means of achieving greater ERK1/2 signaling in a highly specific manner (Supplemental Table 3). Regardless of the temporal induction of MEK1-ERK1/2 signaling, we observed a shift toward a slow, oxidative phenotype in all muscles examined in this study. The *MyoD-icre* mouse also produced near identical results in promoting the slow fiber-type transition, and because this Cre is active prior to developmental fiber-type specification, it indicates that MEK1-ERK1/2 signaling acts independent of initial fiber-type identity.

Previous research of MAPKs in MD revealed that the effector kinases p38 and JNK1 are upregulated and even contributory to the disease pathogenesis. The myofiber-specific overexpression of MKK6, the upstream kinase dedicated to the regulation of p38, leads to a dystrophic-like pathology (21). Conversely, the loss of p38 protected myofibers from necrosis in *Sgcd*^–/–^ mice by decreasing the activity of Bcl-2–associated X (Bax), a proapoptotic protein and p38 substrate. Kolodziejczyk et al. demonstrated greater JNK1 activity in the context of MD that was associated with an increased interaction between JNK1 and the transcription factor nuclear factor of activated T-cells c1 (NFATc1), thus resulting in the nuclear exclusion of NFATc1. Introducing a JNK1 inhibitory protein directly into myofibers of MD mice resulted in healthy fibers resembling those from non-diseased controls (48). In the present study, we demonstrate that the myofiber-specific increase in ERK1/2 levels attenuated the histopathology in *Sgcd*^–/–^ mice and improved their functional capacity. The structural integrity of the sarcolemma was protected from damage in *Sgcd*^–/–^ mice with increased ERK1/2 signaling compared with controls. The reduction in IgM-positive fibers was associated with an upregulation in dystrophin expression and the dystrophin autologous homologue utrophin A. The significance of utrophin A expression was first demonstrated by Tinsley et al. using transgenic mouse models expressing full-length utrophin A, which prevented MD when crossed into the *mdx* background (49). Oxidative skeletal muscles express more utrophin mRNA levels because of enhanced mRNA stability regulated by a calcineurin/NFAT signaling-dependent mechanism (41).

Calcineurin is a calcium/calmodulin-regulated protein phosphatase that, once activated, can directly dephosphorylate members of the NFAT transcription factor family (50). Transgenic mice expressing an active form of calcineurin in skeletal muscle display a fast-to-slow fiber-type switch (43) and when crossed with *mdx* mice, improve the disease pathology (44). Conversely, the loss of calcineurin A α/β isoforms leads to a downregulation of the slow, oxidative program in skeletal muscles (51). Cross-talk between MEK1-ERK1/2 and the calcineurin/NFAT signaling pathways has previously been established in the heart where these pathways interact (52). Immunoprecipitation experiments using protein lysate from cardiomyocytes revealed the formation of a protein complex involving MEK1-ERK2-calcineurin-NFAT. We show that calcineurin levels are increased in skeletal muscles expressing more MEK1-ERK1/2 protein, suggesting that the same protein complex is likely formed in this tissue as well.

We have shown that increased MEK1-ERK1/2 signaling is directly responsible for programming the fast-to-slow fiber-type program in vivo on many levels. Going forward, this knowledge could be exploited for therapeutic or muscle performance advantage. Further investigation is warranted as to whether the pharmacological activation of ERK1/2 signaling could alleviate the dystrophic pathology. To our knowledge, there is no known pharmacological compound dedicated to the activation of MEK1 or ERK1/2. However, we have demonstrated that the loss of DUSP6 and DUSP8 leads to an oxidative fiber-type shift in skeletal muscles, suggesting an alternative approach with a small molecule to inhibit these phosphatases in affecting MD.

## Methods

### Animal models.

Animal experiments performed in the study were approved by the Institutional Animal Care and Use Committee of the Cincinnati Children’s Hospital Medical Center. All mice were maintained on the C57BL/6 genetic background. Mice did not undergo randomization because they were genetically identical and many groups were from the same litters, as well as matched for age and sex ratio. Both male and female mice were used in an equal ratio, and no sex-specific differences were observed. Experiments with mice were performed in a blinded manner where possible.

Mouse lines used in the study were: *Mapk3^–/–^* (ERK1) mice (30), *Mapk1^f/f^* (ERK2) mice (29), *Dusp6^–/–^* (34), *Dusp8^–/–^* (35) mice, *Sgcd^–/–^* mice (40), and mice harboring a Cre-dependent constitutively active MEK1 cDNA inserted into the *Rosa26* locus (53) (Jax, 012352). *Mapk1^f/f^* animals and those with the constitutively active MEK1 cDNA containing-allele were crossbred with mice expressing Cre recombinase under the control of the myosin light chain 1/3 (*Myl1*-cre) genomic locus (23). In addition, we crossed the mice with the constitutively active MEK1 cDNA allele with either *MyoD-icre* gene-targeted animals (31) or with Ska-MCM–transgenic mice (32), the latter of which has the MerCreMer tamoxifen inducible encoding cDNA (54) driven by the human α-skeletal actin promoter (55). Tamoxifen was administered to *Rosa26*-*MEK1^Ska–MCM^* and controls via intraperitoneal injections at 2 months of age for 5 consecutive days (75 mg/kg) using pharmaceutical-grade tamoxifen (MilliporeSigma) dissolved in corn oil (MilliporeSigma). Subsequently, the mice were fed a diet containing 400 mg/kg tamoxifen citrate (Envigo, TD.55125) for the duration of the study. *Mapk3^–/–^* and *Mapk1^f/f^* mice were crossbred with Myh7-cre–transgenic mice (6) to delete *Mapk1* (ERK2) specifically in slow twitch myofibers. Mice were sacrificed by isoflurane inhalation followed by cervical dislocation.

### Immunofluorescence.

Histological cross sections (8 μm) were collected from skeletal muscles using a cryostat and maintained in blocking solution (10% goat serum diluted in PBS) for 30 minutes in a humid chamber. Slides were stained overnight at 4°C with PBS containing either or a combination of anti-Myh7 (BA-F8, 1:50, Developmental Studies Hybridoma Bank [DSHB]) primary antibody, anti-Myh2 primary antibody (SC-71, 1:20, DSHB), anti-Myh4 primary antibody (BF-F3, 1:10, DSHB) and with anti-laminin antibody (1:200, L9393, MilliporeSigma) to delineate myofiber outlines present in a muscle section. Immunoglobulin M (IgM) primary antibody conjugated to FITC (1:300, SAB4700348, MilliporeSigma) was used to identify myofibers with compromised membrane integrity. Primary antibodies were visualized using Alexa Fluor 568 goat anti-mouse IgG2b (Invitrogen), Alexa Fluor 488 goat anti-mouse IgG2b (Invitrogen), Alexa Fluor 674 anti-mouse IgG1, Alexa Fluor 568 IgGm, and Alexa Fluor 405 or 568 goat anti-rabbit IgG secondary antibodies diluted 1:500 in PBS. Immunofluorescence images were captured using a Nikon Eclipse Ti microscope. Entire muscle sections were analyzed for type I positive fibers. All experiments were performed in a blinded fashion whereby the experimenter was only made aware of the genotypes after the quantification was performed.

### Western blotting.

Muscles were homogenized and lysates prepared as previously described (21). Proteins were resolved by SDS-PAGE, transferred to Immobilon-FL membranes (MilliporeSigma) and incubated overnight with antibodies against, calcineurin A (1:500, EP1669Y, Abcam), dystrophin (1:200, PA1-21011, Thermo Fisher Scientific), ELK-1 (1:100, E-5, Santa Cruz Biotech), p-ELK1 (1:200, B-4, Santa Cruz Biotech), EGR1 (1:500, 15F7, Cell Signaling Technology), ERK1/2 (1:2000, 137F5, Cell Signaling Technology), GAPDH (1:500 000, 10R-G109A, Fitzgerald), MEK1/2 (1:1000, 9122, Cell Signaling Technology), MSK1 (1:500, C27B2, Cell Signaling Technology), p-MSK1 Thr581 (1:300, 9595, Cell Signaling Technology), p-MSK1 Ser376 (1300, 9591, Cell Signaling Technology), RSK (1:1000, 32D7, Cell Signaling Technology), p-RSK1 Ser380 (1:500, 934, Cell Signaling Technology), p-RSK1 Thr359 Ser363 (1:500, 9344, Cell Signaling Technology), δ-sarcoglycan (1:500, EPR8706, Abcam), β-tubulin (E7, 1:200, DSHB), and utrophin A (1:500, 8A4, Santa Cruz Biotech). Membranes were then incubated with IRDye secondary antibodies (1:6000, LI-COR Biosciences) and visualized using an Odyssey CLx imaging system (LI-COR Biosciences; see complete unedited blots in the supplemental material).

### Reverse-transcriptase PCR.

RNA was extracted from flash frozen EDL and gastrocnemius muscle using the RNeasy kit (Qiagen) according to the manufacturer’s protocol. Total RNA was reverse transcribed using random oligo-dT primers and Superscript Reverse Transcriptase (Thermo Fisher Scientific).

qPCR was performed using SsoAdvanced SYBR Green (Bio-Rad, 6090), and GAPDH expression was used for normalization. All experiments were performed in duplicate. The following primer sets were used to identify transcripts: Atp2a2, 5′-GAGAACGCTCACACAAAGACC, 5′-CAATTCGTTGGAGCCCCAT; Myh7, 5′-ACTGTCAACACTAAGAGGGTCA, 5′-TTGGATGATTTGATCTTCCAGGG; Tnnc1, 5′-GCGGTAGAACAGTTGACAGAG, 5′-CCAGCTCCTTGGTGCTGAT; Tnni1, 5′-ATGCCGGAAGTTGAGAGGAAA, 5′-TCCGAGAGGTAACGCACCTT; Tnnt1, 5′-CCTGTGGTGCCTCCTTTGATT, 5′-TGCGGTCTTTTAGTGCAATGAG. GAPDH was used as an internal control 5′-TGACCACAGTCCATGCCATC and 5′-GACGGACACATTGGGGGTAG. Statistical analyses were performed using data generated by calculating the ΔCt (the difference in the Ct value between the gene of interest and the GAPDH Ct value). Fold changes were calculated using the ΔΔCt method and the fold-change ranges were calculated using error propagation.

### Pathological indices.

Histological cross sections (8 μm) were collected from skeletal muscles using a cryostat and stained for H&E or picrosirius red to assess myofibers with centrally located nuclei and fibrosis, respectively. The number of myofibers with centrally located nuclei was quantified from two ×10 micrographs taken from histological sections of the quadriceps at mid-belly. Fibrosis was quantified from two ×10 pictures taken from histological sections of the quadriceps. Quantification of fibrotic area was calculated using the ImageJ analysis software. The minimal Feret’s diameter was determined using ImageJ. All analyses were performed in a blinded fashion whereby the experimenter was only made aware of the genotypes following proof of quantification. Fresh histological muscle sections collected from frozen samples were submitted to the Cincinnati Children’s pathological core for SDH staining.

### Measurement of basal metabolic parameters.

To monitor mouse oxygen consumption during exercise, a sealed motorized treadmill was used. The treadmill had adjustable speed and inclination settings and was equipped with an electric shock-delivering grid. During the acclimatization period, the treadmill was set at its slowest speed (3 m/min) for 10 minutes followed by an exercise period (25 m/min) lasting 20 minutes. Electric shock intensity was set to 1 mA and the inclination was set to 5%. Fresh air was delivered with an electric pump and experiments were performed at 23°C. Gas samples from the treadmill chamber were collected every 15 seconds and analyzed by the Oxymax System Metabolic and Telemetric Modular Treadmill (Columbus Instruments, model 1012R-1) for measurement of VO_2_ and VCO_2_. Respiratory exchange ratio was calculated as VO_2_/VCO_2_ with the CLAMS data examination tool (Columbus Instruments).

### In situ TA isometric force measurement.

Mice were anesthetized with isofluorane and a 1.5-cm incision was made along the femur exposing the sciatic nerve that allowed for the identification of the tibia branch of this nerve. Both the tibia branch and the sciatic nerve were cut, leaving the peroneal branch of the nerve intact for stimulation. The skin running from the ankle to the thigh was cut and removed to expose the TA muscle. The mouse was then placed in a supine position on the muscle testing apparatus while a radiant lamp was used to maintain the animal’s body temperature. The knee was immobilized at the limb clamp (Aurora Scientific) and the foot was fixed using surgical tape. The lower third of the TA muscle was dissected off the tibia and the tendon was resected and attached to the lever arm using silk suture to measure force production (Aurora Scientific, 305C). The sciatic nerve was then placed on 2 needle electrodes and stimulated to determine the maximal isometric twitch force with 0.2-ms pulses at 50 mA. The muscle was stretched until the optimal length was reached that allowed for maximal force production. Next, the maximal isometric tetanic force was measured by increasing the frequency from 25 Hz to 200 Hz. The duration of the contractions was set at 350 ms and a contraction was elicited every 2 minutes.

Muscle fatigue was achieved by repeated maximum isometric tetanic contractions at a rate of 1 contraction every 3 seconds for 100 contractions. The maximal tetanic force was evaluated after each contraction during the fatigue protocol. In addition, we measured the maximal tetanic force at 2 minutes and 5 minutes following the fatigue protocol to assess postfatigue recovery. Data were collected and analyzed using the Dynamic Muscle Control Analysis program and software (Aurora Scientific). Muscle weight and length were measured to calculate the physiological cross-sectional area using the following formula: [muscle mass (g)/1.06 (fiber density) × muscle length (cm) × 0.6 (muscle length to fiber length ratio)]. To normalize the maximal force production, we calculated the specific force by dividing the absolute tetanic force by the physiological cross-sectional area of the TA muscle.

### Treadmill running.

Mice were subjected to forced downhill treadmill running using a ramping speed protocol as previously described (21). Mice were run until exhaustion or until the entire protocol was completed.

### Transmission electron microscopy.

Samples were processed, stained, and imaged as previously described (56). Briefly, freshly dissected TA muscles were immersed in relaxing buffer (0.15% sucrose, 5% dextrose, 100 mM KCl in PBS) before being fixed (3.5% glutaraldehyde, 0.15% sucrose in 0.1 M sodium cacodylate pH 7.4) and post-fixed in 1% OsO_4_ (in water). Samples were then embedded using Epoxy resin, sectioned, and counterstained for visualization. Images were captured using a Hitachi 7600 transmission electron microscope connected to an AMT digital camera.

### Statistics.

A 1-way ANOVA was used to determine whether there was a significant difference in experiments with more than 2 groups; a Tukey’s post hoc test was performed to compare individual groups (Prism Software). Significant differences between 2 groups were determined using both 1- and 2-tailed unpaired Student’s *t* tests (Stats Plus Software). Significance was set at *P* < 0.05 for all experiments. All results are presented as mean and the error bar represents the SEM.

### Study approval.

All experiments involving mice were approved by the IACUC at Cincinnati Children’s Hospital Medical Center, approval number IACUC 2016-0069.

## Author contributions

JGB and JDM designed the project and wrote the manuscript. JGB, VP, TS, DL, XF, KG, and MS performed experiments. JGB, TS, and JDM analyzed the data and SS supervised TS.

## Supplementary Material

Supplemental data

## Version 1 04/09/2019

In-Press Preview

## Version 2 05/16/2019

Electronic publication
