# Supplementary material for: ERK1/2 signaling induces skeletal muscle slow fiber-type switching and reduces muscular dystrophy disease severity
Source: JCI Insight. 2019 May 16;4(10):e127356. doi: 10.1172/jci.insight.127356 (PMC6542606; doi:10.1172/jci.insight.127356)
Supplement: Supplemental data [file jciinsight-4-127356-s089.pdf]

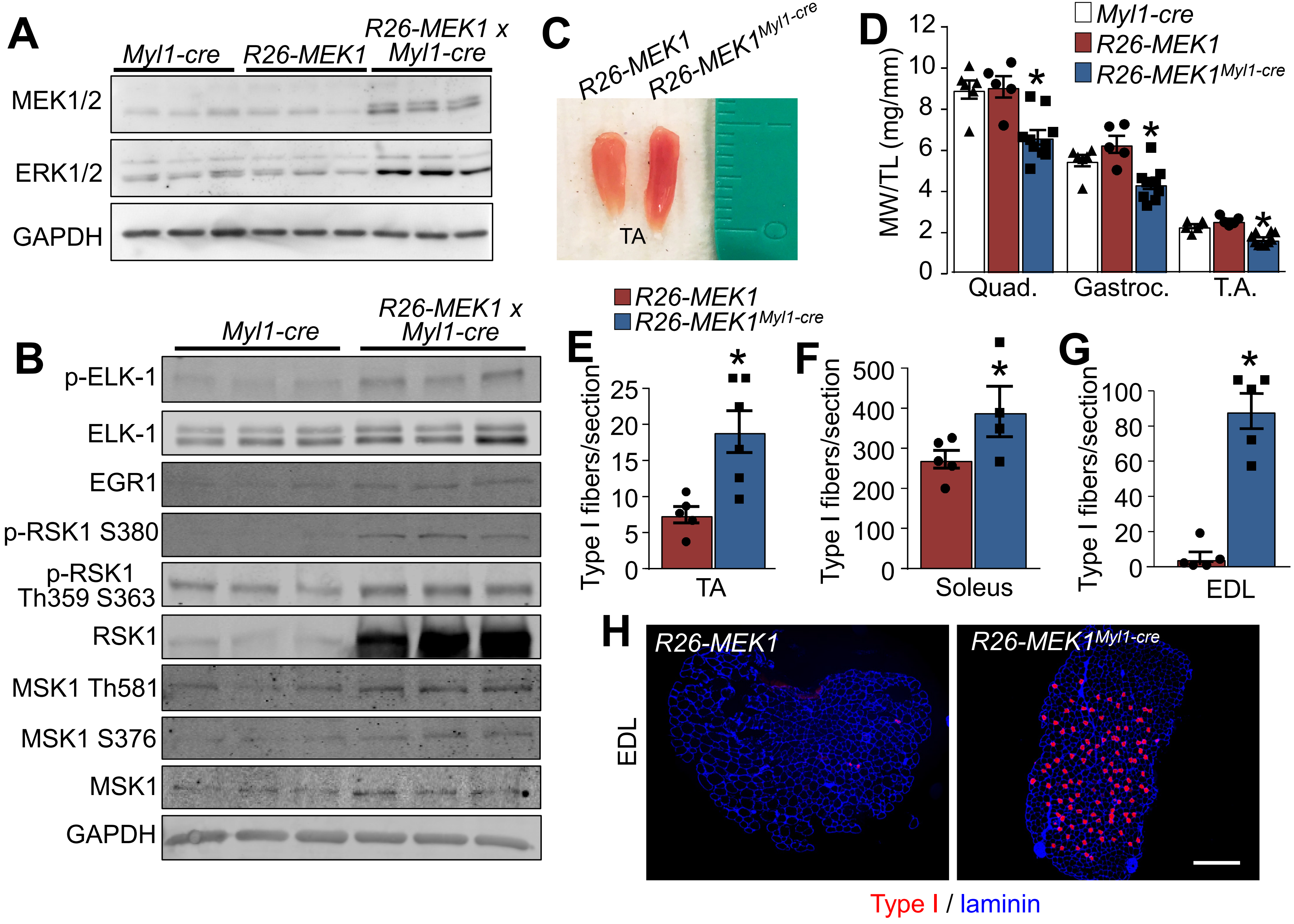
A
Myl1-cre
R26-MEK1
R26-MEK1 x Myl1-cre
MEK1/2
ERK1/2
GAPDH
B
Myl1-cre
R26-MEK1 x Myl1-cre
p-ELK-1
ELK-1
EGR1
p-RSK1 S380
p-RSK1 Th359 S363
RSK1
MSK1 Th581
MSK1 S376
MSK1
GAPDH
C
R26-MEK1
R26-MEK1Myl1-cre
TA
D
Myl1-cre
R26-MEK1
R26-MEK1Myl1-cre
MW/TL (mg/mm)
Quad.
Gastroc.
T.A.
E
R26-MEK1
R26-MEK1Myl1-cre
Type I fibers/section
TA
F
Type I fibers/section
Soleus
G
Type I fibers/section
EDL
H
R26-MEK1
R26-MEK1Myl1-cre
EDL
Type I / laminin

Supplemental Figure 1. MEK1-ERK1/2 signaling increases the number of type I fibers analyzed at 2 months of age.

(**A**) Western blot analysis for total MEK1/2 and total ERK1/2 protein using lysate from the gastrocnemius muscle from 2 month-old mice of the indicated genotypes. n = 3 for both groups. GAPDH is shown as loading control. (**B**) Western blot analysis for protein levels of known ERK1/2 substrates from gastrocnemius protein preparations from 6 month-old mice of the indicated genotypes. n = 3 for both groups. GAPDH is shown as a loading control. (**C**) Representative image of the tibialis anterior (TA) muscles from 2 month-old *Rosa26-MEK1* and *Rosa26-MEK1*$^{Myl1\text{-}cre}$ animals. (**D**) Muscle-weight normalized to tibia-length (MW/TL) at 2 months of age from mice of the indicated genotypes. Muscles analyzed are shown. n = 6 (*Myl1-cre*), n = 5 (*Rosa26-MEK1*), and n = 8 (*Rosa26-MEK1*$^{Myl1\text{-}cre}$). One-way ANOVA with Tukey's multiple comparisons test was used for statistical analysis. *P < 0.05 versus controls. (**E-G**) Quantitation of type I fibers in sections from the TA muscle (**E**), the soleus muscle (**F**) and the EDL muscle (**G**) of *Rosa26-MEK1* and *Rosa26-MEK1*$^{Myl1\text{-}cre}$ mice at 2 months of age. n = 5 (TA, Soleus and EDL: *Rosa26-MEK1*), n = 6 (TA: *Rosa26-MEK1*$^{Myl1\text{-}cre}$), n = 4 (Soleus: *Rosa26-MEK1*$^{Myl1\text{-}cre}$), and n = 5 (EDL: *Rosa26-MEK1*$^{Myl1\text{-}cre}$). Statistical significance was determined using a t-test. *P < 0.05 versus *Rosa26-MEK1*. (**H**) Representative immunohistochemical stained images from the EDL muscles showing Myh7 (red) positive type I fibers and laminin expression (blue) used to identify all myofibers present in sections from mice of the indicated genotypes. Scale bars = 500 µm.

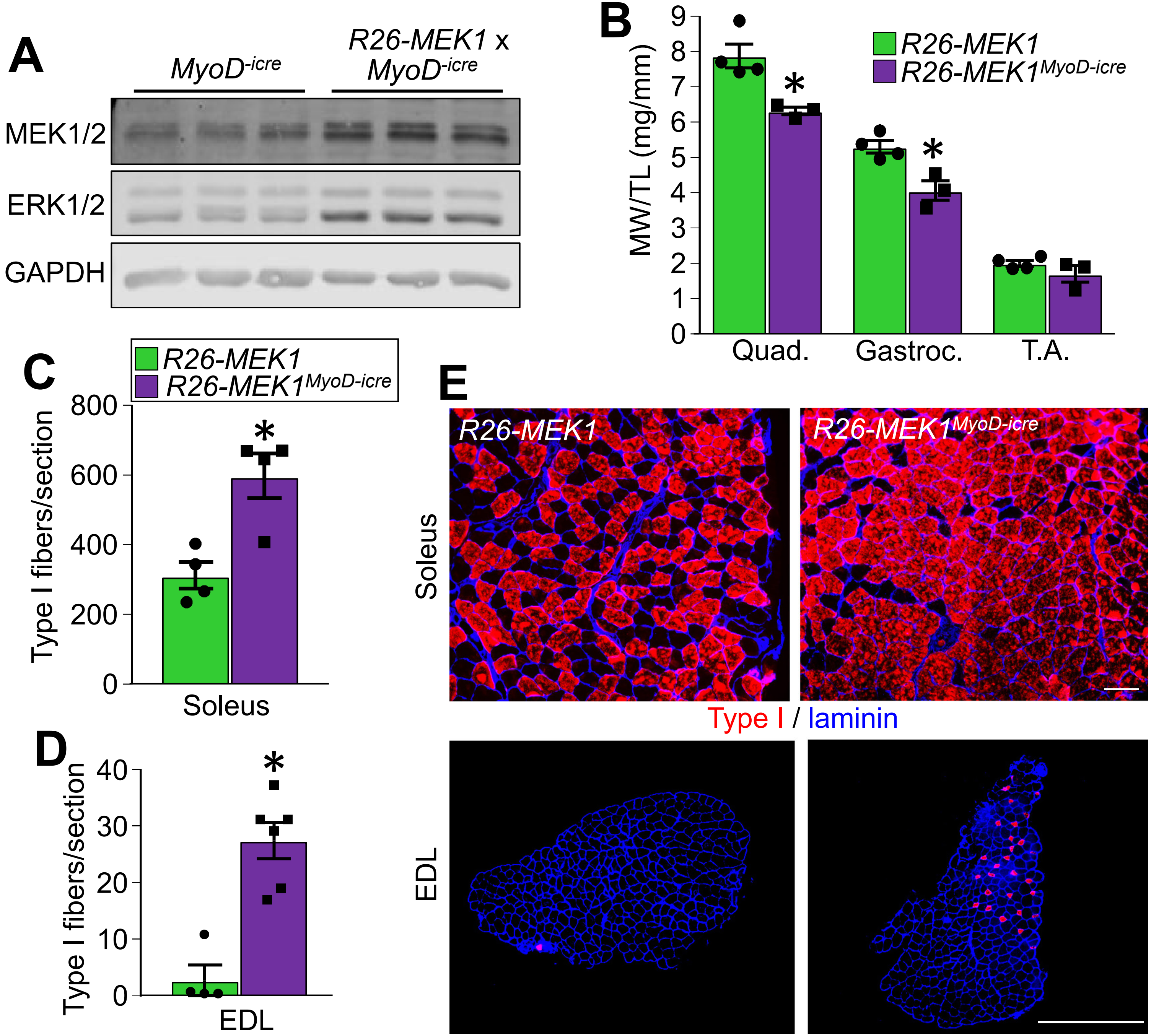
A
MyoD-icre
R26-MEK1 x MyoD-icre
MEK1/2
ERK1/2
GAPDH
B
MW/TL (mg/mm)
R26-MEK1
R26-MEK1MyoD-icre
Quad.
Gastroc.
T.A.
C
Type I fibers/section
Soleus
D
Type I fibers/section
EDL
E
R26-MEK1
R26-MEK1MyoD-icre
Soleus
Type I / laminin
EDL

Supplemental Figure 2. Expression of activated MEK1 in muscle progenitors leads to a slow oxidative phenotype. (**A**) Western blot analysis for total MEK1/2 and total ERK1/2 proteins using lysate from the gastrocnemius muscle from 6 month-old mice of the indicated genotypes. n = 3 per group. GAPDH is shown as loading control. (**B**) MW/TL at 6 months of age for the Quad, Gastroc, and TA muscles from mice of indicated genotypes. n = 4 (*Rosa26-MEK1*) and n = 3 ($Rosa26\text{-}MEK1^{MyoD\text{-}icre}$). (**C-D**) Type I fiber quantification in a histological section taken at the mid-belly from the soleus (**C**) and the EDL (**D**) muscle from 6 month-old mice with indicated genotypes. n = 4 (Soleus and EDL: *Rosa26-MEK1*), n = 4 (Soleus: $Rosa26\text{-}MEK1^{MyoD\text{-}icre}$) and n = 6 (EDL: $Rosa26\text{-}MEK1^{MyoD\text{-}icre}$). (**E**) Representative images from immunostained sections for type I fibers (red) and laminin (blue) from the soleus and EDL muscle from mice of indicated genotypes. Scale bars = 500 µm. A t-test was used to analyze groups for statistical significance. *P < 0.05 versus *Rosa26-MEK1*. Data are represented as the mean and the error bars represent SEM.

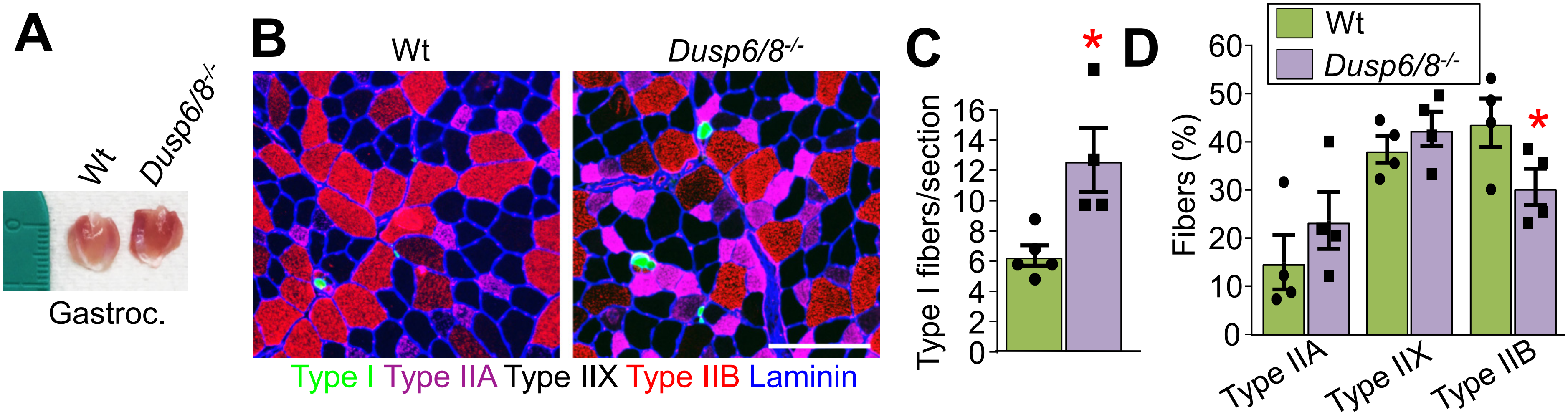

Supplemental Figure 3. Loss of *Dusp6* and *Dusp8* leads to fast-to-slow fiber-type switch.

(**A**) Representative image of the gastrocnemius muscle from 4 month-old mice of the indicated genotypes. (**B**) Representative histological sections immunostained for Myh7 protein (type I, green), Myh2 (type IIA, purple) and Myh4 (type IIB, red) to identify fiber types and for laminin (blue) to delineate myofiber outlines in histological sections from TA muscles of mice of the indicated genotypes. Type IIX fibers were identified by the absence of staining. Scale bar = 250 μm. (**C**) Type I fiber quantification in the TA muscle from histological sections taken at the mid-belly from mice of indicated genotypes; n = 4 per group. Data are represented as the mean and the error bars represent SEM. (**D**) Fiber type quantification in the TA muscle of 4 month-old mice of indicated genotypes. Average number of fibers as the percentage of total fibers is plotted, the error bars represent SEM. Significance was determined using a t-test. *$P < 0.05$.

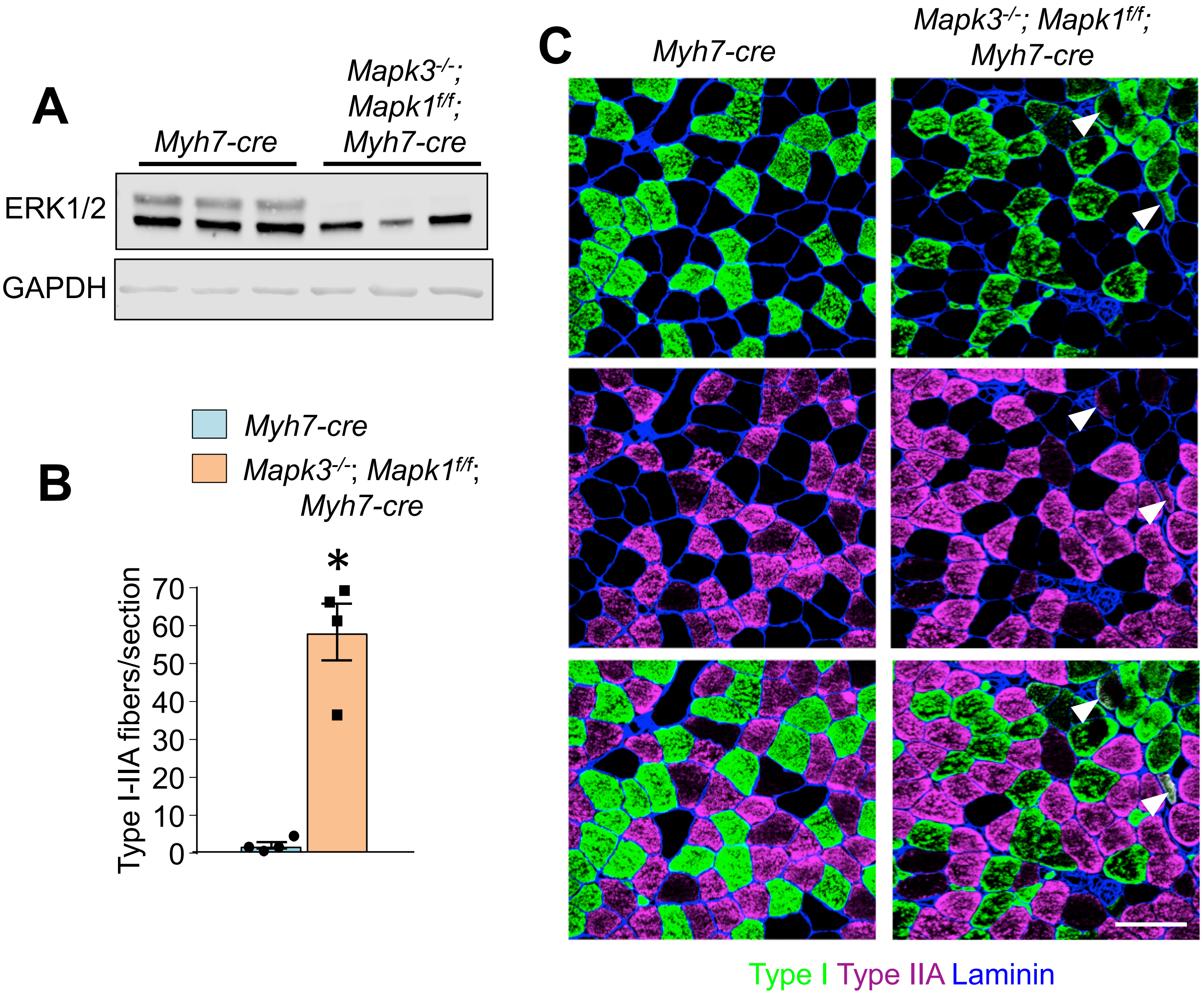

A
Myh7-cre
Mapk3-/-; Mapk1f/f; Myh7-cre
ERK1/2
GAPDH
B
Myh7-cre
Mapk3-/-; Mapk1f/f; Myh7-cre
Type I-IIA fibers/section
70
60
50
40
30
20
10
0
*
C
Myh7-cre
Mapk3-/-; Mapk1f/f; Myh7-cre
Type I Type IIA Laminin

Supplemental Figure 4. ERK1/2 signaling is required for the maintenance of type I fibers in the soleus muscle.

(**A**) Western blot analysis for total ERK1/2 protein levels from the soleus muscle from mice of the indicated genotypes; n = 3 per group. (**B**) Quantification of type I-type IIA hybrid myofibers in histological sections taken at the mid-belly of the soleus muscle from 4 month-old mice of the indicated genotypes; n = 4 per group. Significance was determined using a student's t-test. *P < 0.05. Data are represented as the mean and the error bars are SEM. (**C**) Representative histological sections from the soleus immunostained for the Myh7 protein (Type I, green) and Myh2 protein (Type IIA, purple). Laminin staining (blue) the outline of the myofibers in soleus muscle from mice of the indicated genotypes. The white arrow heads point to hybrid fibers positive for both Myh7 and Myh2 proteins. Scale bar = 100 μm.

Supplemental Table 1. qPCR fold-change ranges for *Rosa26-MEK1*$^{Myl1\text{-cre}}$ samples

	Fold-change range	
Gene of Interest	*Rosa26-MEK1*	*Rosa26-MEK1*$^{Myl1\text{-}cre}$
Myh7	0.2-6.3	18-35
Tnni1	0.3-4	12-30
Tnnc1	0.3-3	11-25
Tnnt1	0.3-3	8-17
Atp2a2	0.4-3	3-8

Abbreviations: Myh7, myosin heavy chain 7; Tnni1, troponin I1; Tnnc1, troponin C1; Tnnt1, troponin T1; Atp2a2, sarcoplasmic/endoplasmic reticulum calcium ATPase 2

Supplemental Table 2. qPCR fold-change ranges for *Rosa26-MEK1*$^{\text{SKA-MCM}}$ samples

	Fold-change range	
Gene of Interest	*Rosa26-MEK1*	*Rosa26-MEK1*$^{SKA\text{-}MCM}$
Myh7	0.2-5	1.5-14
Tnni1	0.5-2	2-5
Tnnc1	0.5-2	2-4
Tnnt1	0.5-2	1-3
Atp2a2	0.4-3	2-9

Abbreviations: Myh7, myosin heavy chain 7; Tnni1, troponin I1; Tnnc1, troponin C1; Tnnt1, troponin T1; Atp2a2, sarcoplasmic/endoplasmic reticulum calcium ATPase 2

Supplemental Table 3. Summary of mouse models and observed phenotype

Mouse model		Observed phenotype
Type	Name and brief description	
Gain of function	*Rosa26-MEK1*$^{Myl1\text{-}cre}$<br>- Myofiber-specific expression of a constitutively active MEK1 cDNA (*Map2k1* gene product)	- Increase in type I fibers<br>- Decrease in muscle weights<br>- Increase in slow oxidative genes<br>- Increase in mitochondria<br>- Increase in oxygen consumption<br>- Increase in fatigue resistance
Gain of function (ERK2)/Loss of function (ERK1)	*Mapk3*$^{-/-}$ *x Rosa26-MEK1 x Myl1-cre*<br>- Myofiber-specific expression of a constitutively active MEK1 cDNA on an *Mapk3*$^{-/-}$ background	- Decrease in muscle weights<br>- Increase in type I fibers
Gain of function (ERK1)/Loss of function (ERK2)	*Mapk1*$^{f/f}$ *x Rosa26-MEK1 x Myl1-cre*<br>- Myofiber-specific expression of a constitutively active MEK1 cDNA on a myofiber-specific *Mapk1*$^{-/-}$ background. The Cre driver is used to recombine both the *Rosa26-MEK1* and the *Mapk1*$^{f/f}$ alleles	- No change in muscle weights<br>- No change in the number of type I fibers
Gain of function	*Rosa26-MEK1*$^{MyoD\text{-}icre}$<br>- Expression of a constitutively active MEK1 cDNA in muscle progenitors leading to an increase in MEK1 expression in all myofibers irrespective of fiber-type	- Decrease in muscle weights<br>- Increase in type I fibers
Gain of function	*Rosa26-MEK1*$^{Ska\text{-}MCM}$<br>- Myofiber-specific inducible expression of a constitutively active MEK1 cDNA	- Decrease in muscle weights<br>- Increase in type I fibers<br>- Increase in slow oxidative genes
Loss of function	*Dusp6/8*$^{-/-}$<br>- Global deletion of *Dusp6* and *Dusp8* leading to increased ERK1/2 activity	- Increase in type I fibers<br>- Decrease in type IIB
Loss of function	*Mapk3*$^{-/-}$ *Mapk1*$^{fl/fl\text{-}Myh7\text{-}cre}$<br>- Slow-fiber-specific deletion of *Mapk1* on *Mapk3*$^{-/-}$ background	- Increase in type I-IIA hybrid fibers
Gain of function	*Rosa26-MEK1*$^{Myl1\text{-}cre}$ *Sgcd*$^{-/-}$<br>- Myofiber-specific expression of a constitutively active MEK1 cDNA on a *Sgcd*$^{-/-}$ background<br>- *Sgcd*$^{-/-}$ mice are a model of limb-girdle muscular dystrophy type 2F	- Increase in slow-oxidative muscle<br>- Decrease in myofibers with centrally located nuclei<br>- Decrease in fibrosis<br>- Increase in running performance<br>- Decrease in the number of IgM positive fibers<br>- Increase in utrophin and dystrophin expression levels
